# Penile Rehabilitation and Treatment Options for Erectile Dysfunction Following Radical Prostatectomy and Radiotherapy: A Systematic Review

**DOI:** 10.3389/fsurg.2021.636974

**Published:** 2021-03-02

**Authors:** Melianthe Nicolai, Ahmet Urkmez, Selcuk Sarikaya, Mikkel Fode, Marco Falcone, Maarten Albersen, Murat Gul, Georgios Hatzichristodoulou, Paolo Capogrosso, Giorgio Ivan Russo

**Affiliations:** ^1^Urology Department, The Netherlands Cancer Institute (NKI), Amsterdam, Netherlands; ^2^Urology Department, Diakonessenhospital, Utrecht, Netherlands; ^3^Haydarpasa Numune Training and Research Hospital, Istanbul, Turkey; ^4^Gulhane Training and Research Hospital, Ankara, Turkey; ^5^Urology Department, University of Copenhagen, Copenhagen, Denmark; ^6^Urology Department, Molinette Hospital, Turin, Italy; ^7^Urology Department, University Hospitals Leuven, Leuven, Belgium; ^8^School of Medicine, Selcuk University, Konya, Turkey; ^9^Martha-Maria Hospital Nuremberg, Nuremberg, Germany; ^10^Vita-Salute San Raffaele University, Milan, Italy; ^11^Urology Department, University of Catania, Catania, Italy

**Keywords:** penile rehabilitation, erectile dysfunction, phosphodiesterase 5 (PDE 5) inhibitors, vacuum devices, intracavernosal injection, pelvic floor therapy

## Abstract

After radical prostatectomy (RP) or radiotherapy (RT) for prostate cancer, erectile dysfunction (ED) is the main complication next to urinary incontinence, affecting quality of life. The pathophysiology of ED after these treatments is believed to include neuropraxia causing reduced oxygenation and structural changes of the tissue in the corpora cavernosa. Next to the option of sparing the nerves during RP, research has been focusing on methods for penile rehabilitation after RP and RT, since it occurs often, even after nerve-sparing techniques were used. In animal studies, the use of phosphodiesterase type 5 inhibitors (PDE5i) after cavernous nerve damage is supported, but results in human studies are contradictory. Non-medical treatment options such as vacuum device therapy, hyperbaric oxygen therapy, yoga, aerobic, or pelvic floor training may be helpful, but evidence is scarce. Clear guidelines for penile rehabilitation are not yet available. However, care and support for ED after RP and RT is highly demanded by a large group of patients, so measures have to be taken even though the evidence is not strong yet. In this systematic review, an overview of the literature for penile rehabilitation and treatment options for ED after RP and RT is provided, using only randomized controlled trials (RCT).

## Introduction

Prostate cancer (PCa) is the second most common cancer among men, its prevalence is increasing, at the moment it accounts for 15% of all and 10% of male cancers (1). Radical prostatectomy (RP) and radiotherapy (RT) are important treatment options for localized PCa, but these techniques lead to erectile dysfunction in many of those receiving them. Erectile function (EF) is, next to urinary symptoms, the main concerns for patients after treatment for PCa (2).

Approximately 45% of patients diagnosed with PCa undergo RP (3); using the nerve-sparing technique leads to lower rates of erectile dysfunction (ED) (4). The pathophysiology causing ED after RP mainly depends on neural injury (5), because even using nerve-sparing techniques, manipulation and physical traction of the nerves may still lead to varying degrees of ED (5).

Intraoperative neurostimulation has been appointed as a useful option, making it easier to save the neurovascular bundle without impairing chances of survival. However, it's use is still not widely spread (3). Several modalities for penile rehabilitation have been described in literature. Mainly involving long term treatment with the established modalities for ED, such as phosphodiesterase-5 inhibitors (PDE5Is) (6–8). Besides this medicinal treatment options, extracorporeal shockwave lithotripsy (ESWT) has been described as a potential option for penile rehabilitation while this treatment may stimulate the Schwann cells (9). In addition, as it is clearly known that the pelvic floor is involved in male sexual function, it may be important to consider pelvic floor rehabilitation in the treatment of ED (10). Other modalities for penile rehabilitation and treatment of ED after RP described in this review are penile vibratory nerve stimulation (PVS), intracavernosal injection therapy, hyperbaric oxygen therapy, and aerobic training.

Shortly after RT, ED is seen in ~40% of the patients. This number rises in the first 2 years after RT to 61.5% (11). RT is often combined with neoadjuvant or adjuvant ADT for localized disease, and even a short term of ADT, negatively affects EF as well (12). PDE5i may protect against ED when started directly after RT. Vacuum erectile devices (VED) and even yoga practice have been studied for their effects on EF after RT. In this systematic review, the literature on this topic is evaluated.

## Materials and Methods

This systematic review was performed according to the Preferred Reporting Items for Systematic Reviews and Meta-analysis (PRISMA) statement. Three authors (S.S., A.U., and M.N.) independently searched PubMed, MEDLINE, EMBASE, PsychINFO, OVID, and Web of Science using the following terms: (prostate cancer OR prostate neoplasm OR prostatic neoplasm OR cancer of the prostate OR prostatic cancer OR prostatic cancers OR prostate neoplasms OR prostate cancer OR prostate neoplasms) AND (radiotherapy OR radiotherapy OR radiotherapies OR radiation therapy OR radiation therapies OR radiation treatment OR radiation treatments OR targeted radiotherapies OR targeted radiotherapy OR targeted radiation therapy OR targeted radiation therapies OR radical prostatectomy) AND (erectile dysfunction OR erectile dysfunction OR male sexual impotence OR male impotence OR impotence OR impotence).

Search criteria were limited to full-text English articles. Only randomized controlled trials (RCT) were included. All relevant papers from 2000 to 2020 were retrieved. References of selected articles and international guidelines were hand searched to identify additional reports.

As this systematic review focused on the management of ED after RP and RT in curative setting, studies that did not focus on specific PCa treatments were excluded. Data extraction was independently performed by three authors (S.S., A.U., and M.N.) and was cross checked afterwards. Disagreements were resolved in consultation with the other authors. When two or more studies were reported by the same institution and/or authors in overlapping time periods, the one which was published more recently was included.

## Results

**Figure 1:**
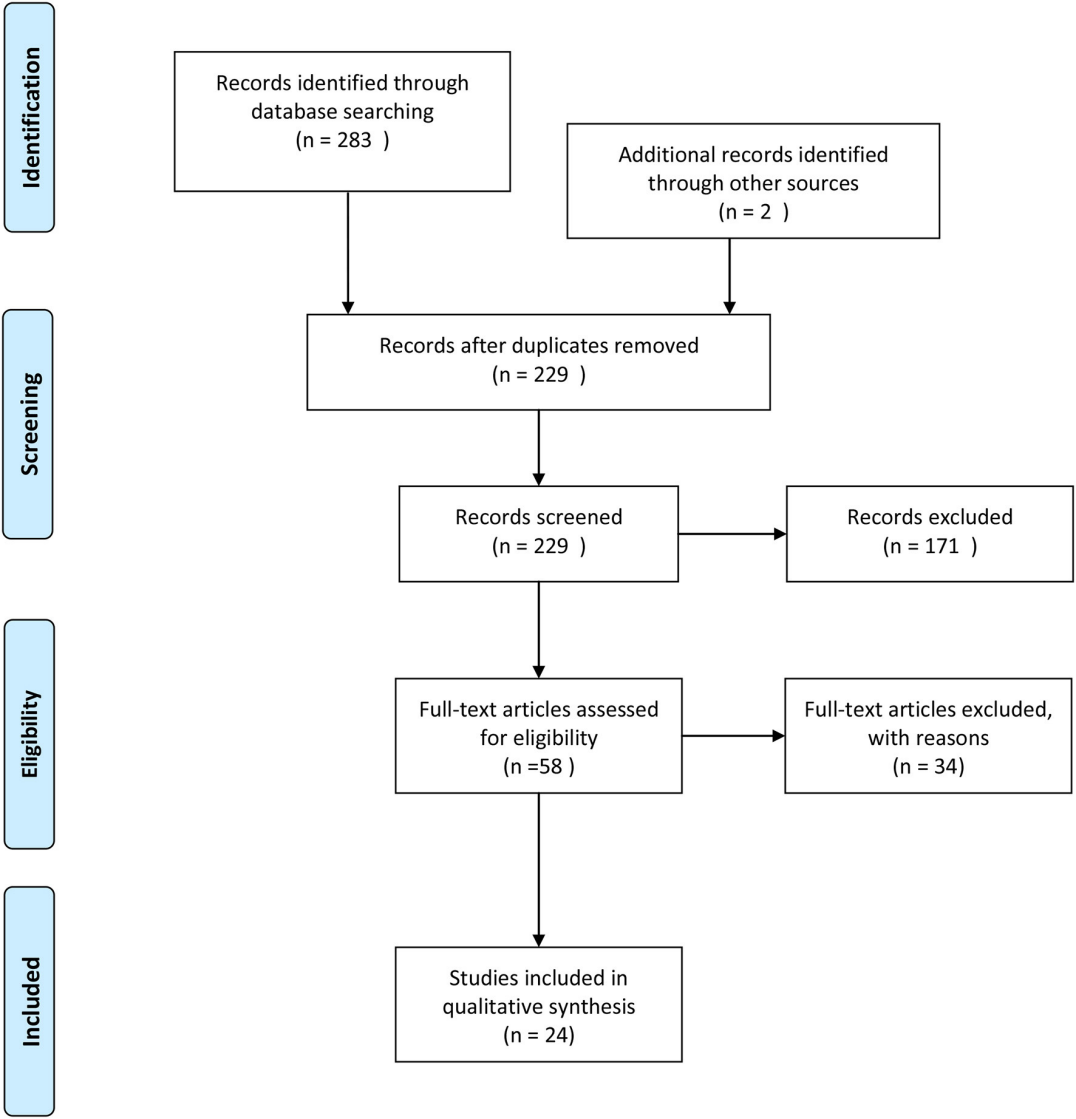
PRISMA flow diagram.

A search of the selected databases revealed 283 articles and two articles identified through other sources. Fifty-six articles were removed as duplicates, and 172 articles were excluded based on inclusion and exclusion criteria. Fifty-seven full-text articles were assessed for eligibility, and 34 articles excluded because they were either systematic reviews, not written in English, or represented non-randomized controlled trials (Figure 1).

## Penile Rehabilitation Following Radical Prostatectomy

We identified 14 RCTs on therapeutic options for ED after RP. Table 1 summarizes the key findings from these studies.

**Table 1:** Overview of RCT about penile rehabilitation after radical prostatectomy.

**References**	**Treatment**	**Sample size**	**Study design**	**Intervention**	**Assessment**	**Outcome measurements**
**PDE5i**						
Padma-Nathan et al. (6)	nsRP start after catheter removal	41 Sildenafil 50 mg 40 sildenafil 100 mg 42 patients placebo	RCT (1:1:1) double-blind Placebo-controlled Multi-center	Either sildenafil 50 or 100 mg nightly or placebo	Erectile function w IIEF (15-item)	Premature closure of study however “erections good enough for satisfactory sexual activity?” in 4% of the placebo group vs. 27% of the sildenafil users.
Montorsi et al. (2)	nsRP start after catheter removal	210 Vardenafil nightly, 210 Vardenafil on demand, 208 placebo	RCT (1:1:1) Phase II trial Double-blind Placebo-controlled Multi-center	9-mo double-blind treatment with a Vardenafil regimen, a 2-mo single-blind washout period + optional 2-mo open-label period, vs. placebo	Erectile function w IEF-EF score (15 item) and Sexual Encounter Profile (SEP) questions 2 and 3	No statistically significant differences among treatment groups in patients with an IIEF-EF score of ≥22 or in SEP3 success rates after washout period. On-demand vardenafil treatment resulted in significantly greater IIEF-EF scores and better SEP3 response rates than placebo over the entire treatment period.
McCullough et al. (13)	nsRP <1 month after surgery	139 intraurethral alprostadil 73 sildenafil citrate 50 mg	RCT (1:1) Prospective, randomized, open label, multicenter	Intraurethral alprostadil nightly or sildenafil 50 mg nightly. After 1-month sildenafil citrate (100 mg) on demand (6 attempts/ month)	Erectile function w IIEF (15-item) and SEP and Erectile Dysfunction Inventory of Treatment Satisfaction and measured stretched penile length	Nightly subtherapeutic intraurethral alprostadil and sildenafil 50 mg both improved penile base and tip rigidity in 24% compared to placebo. The benefit to return of erectile function of nightly sildenafil citrate and subtherapeutic intraurethral alprostadil appears to be comparable within the first year of surgery.
Bannowsky et al. (14)	11 unilateral nsRP 32 bilateral nsRP	23 sildenafil 25 mg nightly 18 no PDE-5 inhibitor	RCT (1:1) prospective, single center	Rigiscan measurement nocturnal erections after catheter removal, patients with preserved nocturnal erections randomized: sildenafil mg/day at night vs. no treatment	Erectile function w IIEF-5 questionnaire at 6, 12, 24, 36, and 52 weeks after NSRP	There was a significant difference in IIEF-5 score and time to recovery of erectile function between the groups (*P* < 0.001), with potency rates of 86 vs. 66%.
Montorsi et al. (15) (REACTT)	nsRP 4 months after surgery	139 tadalafil once daily *143 tadalafil* on demand *141* placebo	RCT (1:1:1) Double-blind, three-arm, parallel-group study, Multicenter, phase 4	9 mo of treatment with tadalafil 5 mg once daily, tadalafil 20 mg on demand, or placebo followed by a 6-wk wash out and 3-mo open-label tadalafil once daily (all patients)	Erectile function w IIEF (15-item), SEP- 3 and penile length	Early initiation of tadalafil (once daily or on demand) had no effect on unassisted erectile function at 10.5 mo after nsRP. Secondary endpoints: IIEF-EF scores ≥22 and SEP-3 significantly higher for tadalafil once daily compared with placebo, exceeding the minimum clinically relevant difference. IIEF-EF and SEP-3 decreased during drugs free washout in all groups and improved during open-label treatment. Penile length loss was reduced vs. placebo in the tadalafil once daily group.
Patel et al. (7)	nsRP 4 months after surgery (data REACTT trial)	139 tadalafil once daily *143 tadalafil* on demand *141* placebo	RCT (1:1:1) double-blind, three-arm, parallel-group study, multicenter, phase 4	9 mo of treatment with tadalafil 5 mg once daily, tadalafil 20 mg on demand, or placebo followed by a 6-wk wash out and 3-mo open-label tadalafil once daily (all patients)	QoL: Expanded Prostate Cancer Index Composite (EPIC-26), Erectile Dysfunction Inventory of Treatment Satisfaction (EDITS), and Self-Esteem and Relationship (SEAR) questionnaires	During dubble blind treatment, IIEF-EF, EPIC sexual domain score, and EDITS score improved significantly with tadalafil dialy vs. placebo but not with tadalafil on demand.
Jo et al. (16)	nsRP	60 patients directly after surgery 60 patients ≥ 3 month after surgery	RCT (1:1) prospective	sildenafil 100 mg (2× /week, for 3 month) immediately after urethral catheter removal recovery or ≥ 3 months after surgery	Erectile function w IIEF (5-item)	Significant improvement of erectile function in early treatment vs. late treatment: 41.4% EF recovery in the early group vs. 17.7% EF recovery in the delayed group at 12 months after surgery.
**INTRACAVERNOSAL INJECTION THERAPY**						
Montorsi et al. (17)	nsRP	30 patients	RCT (1:1) prospective	alprostadil injections 3× /weeks for 12 weeks or observation, 6 mo follow up	sexual history, physical examination, color Doppler sonograph and polysomnographic recording of nocturnal erections	67% recovery of spontaneous erection sufficient for satisfactory sexual intercourse in treatment group vs. 20% in observation group (*p* < 0.01)
**VACUUM DEVICE THERAPY**						
Köhler et al. (18)	Uni or bilateral nsRP	14 patients early intervention protocol with VED 1 month after RP, 14 control group, 6 months after RP using VED	RCT (1:1) Prospective	daily rehabilitation protocol consisting of 10 min/days using the VED without constriction ring, for 5 months. Up visit was 9.5 (6–12) months after RP.	IIEF-5 questionnaire and measurements of penile flaccid length, stretched length, prepubic fat pad, and midshaft circumference before and at 1, 3, 6, 9, and 12 months after RP	IIEF scores higher in early intervention at 3 months (*P* = 0.008) and 6 months (*P* = 0.012), after RP. No significant changes in penile flaccid length, prepubic fat pad, or mid-shaft circumference in either group. Stretched penile length was preserved in early group and decreased by ~2 cm (*P* = 0.013) in late intervention group.
Raina et al. (19)	nsRP and non-nerve-sparing (NNS) RP	74 patients daily VED use for 9 months 35 observation	14 patients Early intervention protocol with VED 1 month after RP, 14 control group, 6 months after RP using VED	RCT (1:1) Prospective	Sexual Health Inventory of Men, IIEF-5, stratified by the NS status. compliance, change in penile length, return of natural erection, and ability for vaginal intercourse	32% in intervention group reported return of natural erections at 9 months vs. 37% in controls with 17% having erections sufficient for vaginal intercourse vs. 11% in controls (significance not mentioned). IIEF-5 score significantly increased after VCD use in both the NS and NNS groups. After a mean use of 3 months, 18% discontinued treatment.
**PVS**						
Fode et al. (20)	nsRP	42 penile vibratory stimulation 31 control	RCT (1:1) Multicenter	Daily penile vibratory stimulation (PVS) Start 1 week before nsRP and for 6 weeks after catheter removal.	Erectile function w IIEF (5-item)	IIEF-5 scores higher in the PVS group at all time points after surgery, but no statistical significance. 12 months after surgery 53% in the PVS group had a IIEF-5 score ≥ 18, vs. 32% in controls (*P* = 0.07).
**TRACOLIMUS**						
Mulhall et al. (21)	nsRP	62 tacrolimus 69 placebo	RCT (1:1) Double-blinded Multicenter	Either Tracolimus or placebo 27 weeks (1 week prior to and 6 months after RP), followed up for 2 years post-RP.	Erectile function w IIEF (15-item) questionnaire	Use of Tracolimus was not associated with improvement in recovery of erectile function after RP.
**LiESWT**						
Baccaglini et al. (22)	Open RP or laparoscopic RP, nerve sparing and non-nervesparing	36 low-intensity extracorporeal shockwave therapy (LiESWT)41 control	RCT (1:1) open-label, 2 parallel arms	Both arms started 5 mg tadalafil/day after removal of catheter., LiESWT received 2,400 shocks/session-week on four different penile regions. The full treatment: 19,200 impulses in 8 weeks.	Erectile function w IIEF (5-item)	Comparing the proportion of patients with an IIEF-5 score ≥17: no significant difference between groups was noted (17.1 vs. 22.2%; *P* = 0.57) 16 weeks after RP.
**HYPERBARIC OXYGEN THERAPY**						
Chiles et al. (23)	nsRP	40 hyperbaric oxygenation (100% oxygen) therapy 43 oxygen enriched air (controls)	RCT (1:1) Double blind, prospective Multicenter	Either exposition 100% oxygen (hyperbaric conditions) or higher pressured air (controls). The primary outcome: erectile function at 18 months.	Erectile function w IIEF (5-item) And EPIC-26	No statistically significant differences between the two groups on any outcome measure.
**AEROBIC TRAINING**						
Jones et al. (24)	nsRP	25 aerobic training 25 usual care (controls)	RCT (1:1) Single center	Aerobic therapy consisted of 5 supervised walking sessions/ week, 30–45 min /session, at 55–100% of VO_2peak_ for 6 mo. Usual care participants maintained their usual exercise levels.	Erectile function w IIEF (15-item)	ED prevalence decreased in both groups from baseline to 6 mo and from baseline to 12 mo, with no significant differences between groups.
**PELVIC FLOOR MUSCLE TRAINING**						
MIlios et al. (25)	Open RP or laparoscopic RP, nerve sparing and non-nervesparing	50 high intensity pelvic floor muscle training 47 control group “usual” pelvic floor muscle training.	RCT (1:1) Single center	Either the usual pelvic floor muscle training of 3 sets/d (controls) Or high intensity pelvic muscle training of 6 sets/d pelvic floor muscle training in standing, both groups Commencing 5 weeks before RP for total of 3 month. Evaluation, 2, 6 and 12 weeks post RP.	Erectile function w IIEF (5-item) Expanded Prostate Cancer Index Composite for Clinical Practice (EPIC-CP)	No statistically significant differences between the two groups on any outcome measure.

*Summary of randomized controlled trials on penile rehabilitation following radical prostatectomy*.

### PDE-5 Inhibitors

To date, five RCTs have evaluated the impact of early usage of PDE5i in men on the recovery of spontaneous erections following nerve-sparing RP (nsRP).

The first of these trials showed that administration of sildenafil every night for 36 weeks, starting 4 weeks after surgery, did significantly increased return of spontaneous erections. However, enrollment was prematurely ended and only 76 men completed the trial because of the fact that the placebo response rate of 25% at blinded interim review, suggested a lack of treatment effect. On the contrary, spontaneous EF [a total score of >8 for questions 3 and 4 of the International Index of Erectile Function (IIEF)] and a positive response to “Were erections good enough for satisfactory sexual activity?” were seen in only 4% of the placebo group vs. 27% (*P* = 0.0156) of the sildenafil group (6).

The second trial was performed by Montorsi et al. (2). They conducted a randomized, double-blind, double-dummy, multicenter study with parallel groups at 87 centers across the world. A total of 628 men after bilateral nsRP were included. One month preoperatively, all had a normal erectile function domain (IIEF-EF) score of more or equal to 26. The primary endpoint: spontaneous erections after wash-out, was not met because no significant differences were observed among treatment groups following washout. IIEF-EF scores of 22 or higher were achieved in 28.9% for placebo, in 24.1% for vardenafil nightly, and in 29.1% for vardenafil on demand. The effect of on-demand use of vardenafil during the double-blind treatment period was chosen as a secondary endpoint. On demand, vardenafil use was associated with significantly higher IIEF-EF scores at all double-blind visits when compared with placebo. It was associated with significantly better sexual encounters over the entire double-blind treatment period as well.

Comparing the usage of an oral PDE5i and intraurethral alprostadil, McCullough et al. (13) performed a prospective, randomized, open-label, multicenter study in American men with normal EF who underwent bilateral nsRP. Subjects started nightly treatment with intraurethral alprostadil or oral sildenafil citrate (50 mg) within 1 month of nsRP and continued for 9 months. No statistical differences were seen for any of the endpoints between these two groups.

Another trial performed by Bannowsky et al. (14), randomized 43 patients into two different follow-up groups. Groups were matched by preoperative IIEF score, age, numbers of nocturnal erections, and status of nsRP and EF before nsRP as evaluated with the IIEF-5. After catheter removal, post-nsRP nightly penile rigidity was measured during Rigiscan®. No medications influencing EF were used during this period. Patients that kept their nocturnal erections as detected during Rigiscan recordings received sildenafil 25 mg/days at night after catheter removal. Controls with a similar number of nocturnal erections were used. Between the groups, a significant difference in IIEF-5 scores and time to recovery of EF was seen at 36 and 52 weeks (both *P* < 0.001). In the sildenafil group, penile erection sufficient for vaginal intercourse were achieved and maintained by 47% at 1 year after nsRP without usage of “on-demand” sildenafil. In the control group, this was 28%. However, the trial did not include a wash-out period, so these findings represented erections with sildenafil treatment vs. erections without sildenafil treatment and not spontaneous erectile function.

The fifth trial evaluating penile rehabilitation with PDE5i was the REACTT trial (15), comparing efficacy of tadalafil 5 mg once daily and tadalafil 20 mg on demand vs. placebo in improving unassisted EF following nsRP when taken over 9 months. EF was defined by the proportion of patients achieving an IIEF score equal or over 22 after 6 weeks of drug-free washout (DFW). The primary endpoint of the trial: quicker return to spontaneous erections was not met. At 10.5 months after nsRP, after DFW, no effect was seen of early initiated tadalafil (once daily or on-demand) on unassisted EF. The authors suggest that the treatment period of 9 months may have been too short to achieve optimal EF recovery. Indeed, recovery rates of EF were low at 10.5 months after nsRP with 25.2% in the tadalafil once daily group, 19.7% in the tadalafil on demand group, and 14.2% with placebo at this time point. Secondary measures included Sexual Encounter Profile question 3 (SEP-3), IIEF-EF scores, and penile length. At the end of the double-blind treatment mean IIEF-EF scores were significantly improved in both the tadalafil on demand and daily groups compared with placebo. For the SEP-3 group, this was seen for tadalafil once daily only. Penile length loss was significantly reduced at 9 months when compared with placebo in the tadalafil once daily group (mean difference 4.1 mm; *P* = 0.032).

In addition to the REACTT trial, Patel et al. (7) secondary outcome measures on QoL and treatment satisfaction were addressed in early post-nsRP patients who participated in the REACTT trial. They evaluated several aspects of QoL using, for example, the Expanded Prostate Cancer Index Composite Short Form (EPIC-26) which addresses sexual, urinary, bowel, and hormonal function, the Erectile Dysfunction Inventory of Treatment Satisfaction (EDITS) and the Self-Esteem and Relationship (SEAR) instrument (assessing patient and partner sexual relationship confidence and self-esteem). EF was measured using the IIEF-EF domain score at three timepoints: baseline (post-nsRP), after double-blind treatment, and after open-label treatment of tadalafil. During double-blind treatment, the IIEF-EF, the EPIC sexual domain score, and the EDITS score did significantly improve with tadalafil daily when compared with placebo. This was not the case for tadalafil on demand. On demand vs. placebo at end of double-blind treatment did not differ. And, after open label treatment tadalafil daily and on demand vs. placebo did not significantly differ either. However, satisfaction with treatment increased significantly in both tadalafil groups. During double-blind treatment, EDITS total scores increased significantly with daily tadalafil (P = 0.05) and on-demand tadalafil (P = 0.041) vs. placebo. Improvement was significant for tadalafil daily vs. placebo only at the end of open-label treatment (P = 0.035).

Timing was showed to be an important factor in penile rehabilitation in the trial of Jo et al. (16), which randomized start of PDE5i directly vs. 3-months post-nsRP. The proportion of patients receiving PDE5i (3× per week sildenafil 100 mg) directly after nsRP during a period of 12 months, achieved full recovery of EF significantly more often than those starting sildenafil in the delayed group, 3 months after the operation (*P* = 0.034).

In conclusion: comparing on-demand and daily tadalafil for the improvement of spontaneous erections after nsRP, no statistical differences were found. However, with the available evidence nothing can be said about the inferiority of tadalafil daily compared to on-demand use. Vardenafil showed good treatment effect for on-demand use, but daily use did not lead to a quicker return to spontaneous erections. Sildenafil used daily did show a positive effect on erectile function, and in one trial, a shorter time to return to spontaneous erections. All PDE5i showed to have beneficial effects on satisfaction with sexual life and especially tadalafil increased quality of intercourse and sexual activity when used daily. Intraurethral alprostadil appears to have similar beneficial effects on penile rehabilitation as sildenafil daily, but the evidence is not bulky enough to draw strong conclusions.

### Intracavernosal Injection Therapy

One trial as perfomed after nsRP, including 30 patients with preoperative good erections that underwent nsRP. They were randomized to alprostadil injections three times per week, for a total of 12 weeks, or observed directly afterwards, starting directly after nsRP. After 6 months, patients were assessed using sexual history, physical examination, color Doppler sonography of the cavernous arteries, and recording of nocturnal erections with polysomnography. In the treatment group, 67% noted recovery of spontaneous erection that was sufficient for sexual intercourse after the 6-months follow-up (in comparison with 20% in the observation group, *P* < 0.01). In the treatment group, all but one patient showed normal erections at nocturnal erection measurement and normal penile hemodynamics with color Doppler sonography. In the observation group 53% showed cavernous veno-occlusive dysfunction and 20% showed cavernous nerve injury.

In conclusion, alprostadil injection therapy 3× per week after nsRP may be an effective treatment to promote recovery rate of spontaneous erections (17).

### Vacuum Erection Device Therapy

In rats after cavernous nerve crushing, vacuum therapy showed to improve intracarvernosal pressure using nerve stimulation and to help preserve penile size in comparison with controls (26, 27). Furthermore, vacuum erectile devices (VED) reduced hypoxia-inducible factors and increased endothelial NO synthase expression and smooth muscle/collagen ratios in these rodent studies (26, 27).

Two randomized trials have tested VED after RP.

The first study from Kohler et al. (18) randomized 28 men in an early or a delayed treatment group after unilateral or bilateral nerve-sparing RP. Starting 4 weeks after surgery, the early treatment group had to use VED daily for two consecutive 5-min intervals (not using the constriction band). The delayed treatment group had to use VED before intercourse (with constriction band). Both groups were offered PDE5Is in addition. Significantly higher IIEF scores were seen in the early treatment group at 3 and 6 months. However, no difference was seen between the groups after 1 year (*P* = 0.75). PDE5I usage did not significantly differ between the groups. Spontaneous erections adequate enough for intercourse were not reported in either group after 1 year follow-up.

The second trial by Raina et al. (19) randomized for daily VED for 9 months after RP (nerve-sparing and non-nerve-sparing) or to no treatment (*n* = 109). Penile constriction bands for intercourse were allowed in the VED group. In the VED group, 20% was excluded because they discontinued the treatment: 55% due to discomfort, 20% due to penile bruising, 17% due to social inconvenience, and 8% due to inability to use the device. After 9 months of follow-up, the mean IIEF-5 score was significantly higher in the treatment group compared with the no-treatment group (16 ± 7.33 vs. 11.1 ± 1.76, *P* < 0.05). From the VED group, 17% reported return spontaneous erections sufficient for intercourse vs. 11% in the no-treatment group; this difference was not significant.

In conclusion, VED may be offered as a supportive measure in the period after RP, increasing chances of successful intercourse, especially when used next to a PDE5i. Conclusions about efficacy on penile rehabilitation cannot be drawn with the current literature.

### Penile Vibratory Nerve Stimulation

Penile vibratory nerve stimulation (PVS) has been shown to stimulate the nerves of the pelvic floor. In up to 90% of men with spinal cord injuries, PVS could induce ejaculation (28). Fode et al. (20) conducted a trial to examine the effect of PVS in the preservation and restoration of EF in conjunction with nsRP. It was hypothesized that PVS in the early postoperative period after RP may stimulate the cavernous nerves through the reflex arch and would help in the restitution from neuropraxia and improvement of long-term EF (20). A total of 68 patients were randomized between daily stimulation at the frenulum from a minimum of 1 week before the surgery and after catheter removal, for 6 weeks after nsRP. At all-time points after surgery, IIEF-5 scores were highest in the PVS group (median 18 points vs. 7.5 points in control group at 12 months, *P* = 0.09) (28); 53% of patients in the PVS group reached IIEF-5 scores of at least 18, compared with 32% of patients in the control group (*P* = 0.07).

In conclusion, there may be a place for PNS in penile rehabilitation; however, more trials are needed to affirm the existing evidence.

### Tacrolimus

Immunophilin ligands are found to have neuroprotective effects in various animal models, including the rat cavernous nerve injury model. This model is believed to be representative of the neural injury that occurs in human beings at the time of RP (29). The immunophilin ligands bind to a series of intracellular signaling proteins: the immunophilins. While found in immune tissue, immunophilins are even more abundant in neural tissue, peripherally as well as centrally (30). Tacrolimus (Prograf, Astellas Pharmaceuticals) is a macrolide immunophilin ligand approved by the Food and Drug Administration for prevention of allograft rejection in liver and kidney transplantation. However, in animal models, tacrolimus was also shown to have neuroprotective and neuroregenerative properties (31).

Mulhall et al. (21) randomized 132 patients with excellent erections prior to RP, receiving tacrolimus or placebo for 27 weeks and followed them up for 2 years post-RP. No differences in IIEF scores were found between these two groups. Other trials evaluating the effects of tacrolimus in EF have not been performed up until to date.

### Low-Intensity Extracorporeal Shockwave Lithotripsy

Evidence has shown improvement of EF after low-intensity extracorporeal shockwave lithotripsy (LiESWT). For example, in patients with vasculogenic ED, it occurs to induce neovascularization and as a consequence to enhancing penile perfusion. This might convert PDE5i non-responders to responders (9). Furthermore, neuroinjury disease models indicated LiESWT to have neuroprotective and neurodegenerative effects (32).

Baccaglini et al. (22) performed the first randomized clinical trial using LiESWT. Ninety-two patients were randomized between application of the LiESWT (2-months period) in the 6th week after bilateral nsRP or in the control group, patients in both groups started tadalafil 5 mg directly after removal of the catheter postoperatively. In the experimental group, the full LiESWT treatment consisted of 19,200 impulses across 2 months. An improvement of EF was seen over the period of the study in the treatment group in which 22.2% of the patients reached an IIEF-5 score of 17 or higher, compared with 17.1% in the control group. However, the difference was not statistically significant. To shine light on the true effect of LiESWT after nsRP, a trial using a larger cohort has to be performed.

In conclusion, no statements can be made for the effect of LiESWT on penile rehabilitation after RP, more RCTs are necessary.

### Hyperbaric Oxygen Therapy

Up until now, just one RCT (23) has been performed randomizing patient post-robot-assisted RP to hyperbaric oxygen therapy or placebo therapy. A total of 109 potent men who underwent robot-assisted bilateral nsRP were randomized to a hyperbaric oxygenation therapy group or a control group. A total of 43 men in the control group (normal air) and 40 in the hyperbaric oxygenation therapy group completed the 18-months follow-up. No statistical differences were seen between the groups looking at the IIEF-5 scores (*P* = 0.611) or any of the other outcome measures. This trial may be limited by the lack of a sham hyperbaric condition in which participants would receive air but at lower pressures than men in the treatment group. Whereas, in this study, controls received air at increased pressure, leading to a partial pressure of oxygen of twice the oxygen available at standard atmospheric conditions.

In conclusion, with only one RCT available, no conclusions can be drawn about the effects of hyperbaric oxygen therapy on return to spontaneous erections after RP.

### Aerobic Training

Erectile dysfunction following nsRP is mainly caused by neuronal damage. However, vascular endothelial cell dysfunction is an important factor as well, leading to impaired penile tissue oxygenation, resulting in smooth muscle apoptosis, fibrosis, and veno-occlusion dysfunction (33). Aerobic training (AT) may be used to improve EF (24). AT leads to a variety of vascular adaptations such as improvements in peripheral artery flow-mediated dilation. Artery flow-mediated dilation provides a good measure of vascular endothelial function. The efficacy of aerobic training (AT) was investigated by a trial conducted by Jones et al., examining AT compared with usual care on ED prevalence in 50 men after nsRP. AT consisted of five walking sessions per week at 55–100% of peak oxygen uptake (VO_2peak_) for 0.5 to 1 h/session following a non-linear prescription (24). ED, measured as an IIEF score under 21, decreased by 20% in the AT group and by 24% in the usual-care group (*P* = 0.406). No significant differences where seen in any of the EF subscales (*P* > 0.05). Although significant differences between groups were observed for changes in flow-mediated dilation and VO_2peak_, favoring AT (24). The authors appointed this lack of significant difference to the different mechanism inducing ED. In heart failure, endothelial-derived nitric oxide (NO) release is the principal contributor to ED, but in the post-RP setting, surgery-induced neuronal injury is the most important contributor. Furthermore, 6 months of AT may be too short to achieve effect on EF.

In conclusion, although aerobic training significantly improves vascular health, AT does not lead to significant differences in erectile function after RP in the first 6 months after surgery.

### Pelvic Floor Therapy

Pelvic floor muscle (PFM) function is shown to be involved in enhancement of penile blood flow. It is well-known that the ischiocavernous muscle facilitates erection and that the bulbocavernous is involved in maintaining it. Blood is blocked from escaping from the corpora carvernosa by contraction of the bulbocavernous muscles by pressing on the deep dorsal vein of the penis. However, literature regarding the role of PFM training in recovery of sexual function after RP is limited. Although, a couple RCTs confirmed a direct link between PFM strength and increased rigidity in erection (34, 35).

Recently, the effects of PFM training on RP-related ED was evaluated in a RCT using a high-intensity vs. “usual-care” PFM training for the pre-rehabilitation of RP-related ED which started 5 weeks prior to surgery (25). Assessments were undertaken using the EPIC-CP and IIEF-5 questionnaires at 5 weeks preoperatively and at 2, 6, and 12 weeks after surgery. As was expected, after RP, a drastic and immediate reduction of EF was seen in both groups. There were no group differences seen in the ED domain scores across the time points. IIEF-5 scores also were similar. This trial was limited in several ways, however, at first by the fact that PFM was performed in both groups. The follow-up was performed only in the first 12 weeks postoperatively in which not much effect on EF is to be expected, and most importantly, no selection was made by the surgical technique used: non-nerve-sparing RP patients were included in both treatment groups together with unilateral and bilateral nsRP patients (25).

In conclusion, a larger randomized trial using clear methodology has to be conducted to assess the true effects of PFM on EF in patient after RP; with the current data, no statements can be made.

## Penile Rehabilitation Following Radiation Therapy

We identified 9 RCTs on therapeutic options for erectile dysfunction after RT. Table 2 summarizes the key findings from these studies.

**Table 2:** Overview of RCT about penile rehabilitation after radiotherapy.

**References**	**Treatment**	**Sample size**	**Study design**	**Intervention**	**Assessment**	**Outcome measurements**
**YOGA**						
Ben-Josef et al. (36)	EBRT (6- to 9- weeks course) for clinical stage I-II PCa	34 patients w yoga, 34 patients w/o yoga	RCT (1:1) Phase II trial	Biweekly yoga interventions (each session 75 min) throughout the 6–9 weeks courses of RT	General quality of life (FACT-G), fatigue (BFI), erectile function (IIEF-5) and IPSS	Less cancer related fatigue w yoga (*p* < 0.001) Higher IIEF-5 scores w yoga (*p* = 0.0333) No significant effect of yoga on IPSS No adverse event w yoga
**PDE5i**						
Harrington et al. (37)	EBRT T1c-3 PCa Completed RT btw 6 months and 3 years prior to study	33 patients w sildenafil-placebo 33 patients w placebo-sildenafil	RCT (1:1) Double-blind Placebo-controlled Cross-over	Either sildenafil 50/100 mg-placebo or placebo-sildenafil 50–00 mg (on demand) Two sexual activity attempts → crossover → 2 attempts	Erectile function w IIEF (15-item)	Significant increase in all domains of IIEF w sildenafil In nearly half of the patients, the improvement in erectile function domain score was more than 5 points.
Ilic et al. (38)	EBRT (11%) BCT (89%) T1c-3 PCa	14 patients w sildenafil 13 patients w placebo	RCT (1:1) Single-center Double-blind Placebo-controlled	Either sildenafil 50–100 mg/days or placebo 1 month after RT for 6 months	Erectile function w IIEF (15-item)	No significant difference in IIEF scores between groups during study and at 2-years follow-up Daily sildenafil 50–100 mg well-tolerated, no serious adverse events
Incrocci et al. (39)	EBRT T1c-3 PCa Completed RT at least 12 months prior to study	30 patients w tadalafil-placebo 30 patients w placebo-tadalafil	RCT (1:1) Double-blind Placebo-controlled Cross-over	Either tadalafil 20 mg-placebo or placebo-tadalafil 20mg (on demand) 6 weeks → crossover → 6 weeks	Erectile function w IIEF (15-item) and Sexual Encounter Profile (SEP) patient dairy	Significant increase in IIEF scores w tadalafil Improvement of erectile function in 67% (tadalafil) vs. 20% (placebo) of patients Successful intercourse w tadalafil (48%) vs. placebo (9%)
Incrocci et al. (40)	EBRT T1c-3 PCa Completed RT at least 6 months prior to study	30 patients w sildenafil-placebo 30 patients w placebo-sildenafil	RCT (1:1) double-blind placebo-controlled cross-over	Either sildenafil 50/100 mg-placebo or placebo-sildenafil 50/100 mg (on demand) 6 weeks → crossover → 6 weeks	Erectile function w IIEF (15-item)	Significant increase in IIEF scores w sildenafil Improvement of erectile function in 45% (sildenafil) vs. 8% (placebo) of patients Successful intercourse w sildenafil (55%) vs. placebo (18%) 90% of patients needed a dose adjustment to 100 mg sildenafil
Pisansky et al. (41)	EBRT (63%) BCT (37%) for Clinical stage I-II PCa	112 patients w tadalafil 5 mg 109 patients w placebo	RCT (1:1) multicenter double-blind placebo- controlled	Either daily tadalafil 5mg or placebo within 7 days after the initiation of EBRT or the date of BCT. Administration was continue for 24 weeks	Erectile function w IIEF (15-item) and Sexual Adjustment Questionnaire (20-item)	No significant difference in any domain of IIEF questionnaire Partners of men treated w tadalafil noted no significant effect on sexual satisfaction and marital adjustment
Ricardi et al. (42)	EBRT cT1-3 PCa	27 patients w on-demand tadalafil 20 mg 25 patients w daily tadalafil 5 mg	RCT (1:1) Phase II trial Not-blinded No control	Either daily tadalafil 5 mg or on-demand tadalafil 20 mg for 12 weeks	Erectile function w IIEF (15-item) and Sexual Encounter Profile (SEP) patient dairy	Significant improvement in all domains of the IIEF in both arms → No difference btw two arms Successful sexual intercourse in nearly 70% of patients in both arms at 3 months Higher treatment compliance w daily tadalafil 5 mg (100 vs. 86%)
Watkins et al. (12)	EBRT cT1b-4 PCa	30 patients w sildenafil-placebo 31 patients w placebo-sildenafil	RCT (1:1) Double-blinded Placebo-controlled Cross-over	Either sildenafil-placebo or placebo-sildenafil 12 weeks → 1 week washout → crossover → 12 week	Erectile function w IIEF (15-item), the Sexual Adjustment Questionnaire (20-item) and Locke's Marital Adjustment test	Only 21% of patients had a treatment-specific response (during sildenafil phase) Significant benefit in erectile response only for patients receiving ≤ 120 days of ADT (*p =* 0.009)
Zelefsky et al. (43)	EBRT BCT	186 patients w sildenafil 93 patients w placebo	RCT (2:1) Double-blinded Placebo-controlled	Either sildenafil (50 mg/days) or placebo Administration was continue for 6 months	Erectile function w IIEF (15-item) questionnaire	Better erectile function and overall satisfaction w sildenafil at 12 months No significant difference in erectile function and IIEF scores at 24 months

*Summary of randomized controlled trials on penile rehabilitation following radiotherapy*.

### PDE-5 Inhibitors

So far, three RCTs have evaluated the effect of early PDE5i usage on EF preservation and recovery of spontaneous erections in men who underwent RT for PCa. Ilic et al. (38), who investigated daily use of sildenafil 50/100 mg after RT (mainly seed brachytherapy), suggested that early use of regular sildenafil does not improve long-term EF, although short-term sexual function may be improved while on medication. However, this study was limited by the small number of patients (14 patients with sildenafil and 13 patients with placebo) and long period of recruiting time. Similarly, Pisansky et al. (41), who conducted a RCT to explore the protective effect of daily tadalafil 5 mg on EF during and after RT, have shown no improvement in EF and sexual satisfaction compared with placebo. Apart from these two RCTs, Zelefsky et al. (43) have reported significant improvement in EF and overall sexual satisfaction at 6th and 12th months with daily use of sildenafil 50 mg during and for half a year after the initiation of RT. However, this positive effect on EF and IIEF scores was no longer significantly prominent at 24 months, yet improvement in satisfaction and desire were persistent at the 24th month (82 vs. 56%). Aside from the rehabilitation studies, daily and on-demand uses of tadalafil has been shown to be effective in improving EF and increasing the ratio of successful sexual intercourse (up to 70% of patients) in two RCTs (39, 42). Higher treatment compliance was observed with daily tadalafil 5 mg usage compared with on-demand tadalafil 20 mg (100 vs. 86%) (42). However, no difference was observed between daily and on-demand use of tadalafil in terms of EF improvement and successful sexual intercourse (42). On-demand uses of Sildenafil 50/100 mg were also shown to be effective in improving EF and increasing the ratio of successful sexual intercourse in four RCTs (12, 37, 40, 43). In the RCTs which use the beginning dose of sildenafil 50 mg, most of the patients (up to 90%) needed a dose adjustment to 100 mg sildenafil (12, 40).

Differences in findings among the studies may also be related to duration of androgen deprivation treatment (ADT) and when ADT had been discontinued and testosterone recovery occurred. Greater benefit in erectile response to sildenafil was shown in patients who received shorter period of ADT (≤4 months) (12). Also, it has been shown that longer time period to start medical therapy after RT is related to poor response to sildenafil (44).

In all of the eight RCTs, sildenafil and tadalafil both appeared well-tolerated with no serious adverse effects even with daily doses of sildenafil 100 mg.

All studies used IIEF questionnaire (15 items) to assess EF. Additionally, two RCTs used the sexual adjustment questionnaire (12, 41) and one RCT used Locke Marital adjustment test (12) to evaluate patient- and partner-reported outcomes. Sexual encounter profile (SEP) patient diary were also used in two RCTs to assess whether sexual attempt was successful or not and the quality of sexual intercourse (39, 42).

In conclusion, overall, daily as well as on demand use of PDE5i are improving EF and satisfaction with sexual intercourse after RT. However, it is still not clear whether PDE5i started shortly after EBRT protects against EF in the first 2 years after RT.

### Vacuum Erectile Devices

The effectiveness of vacuum erectile devices as first-line treatment option of erectile dysfunction is well-established (45). Although, currently, no high-quality evidence exists, ongoing research suggests in addition to early PDE5i, VEDs may be effective in preventing penile shrinkage and preserving the EF in the context of RT (45).

### Yoga Practice During Radiation Therapy

In a RCT (36), biweekly yoga interventions were shown to be feasible and well-tolerated during the course of 6–9 weeks of radiation therapy and effective in improving fatigue and EF. Sixty-eight patients were randomized to yoga and no-yoga cohorts. Twenty-two patients completed all the yoga sessions, whereas 28 patients stayed in no-yoga cohort at the end of the study. Patients who agreed to participate in yoga practice remained unchanged during the RT course, but the control group showed a decrease in sexual function (IIEF score) during the same period. The authors attributed the observed favorable effect of yoga on EF to improved strength of PFM, induction of a relaxation response through nitrite oxide release, and yoga's effect on patients' mental health (36).

## Discussion

Prostate cancer is the most common malignancy among men. PDE5is have been shown to be effective in the treatment of ED after both RP and RT.

Chronic dosing of PDE5i was proposed as a measure to accelerate recovery of return to spontaneous erections after nsRP (7). Nightly and long-term administration of sildenafil did indeed show to increase the return of spontaneous erections (6). The other PDE5i did not show significant increase in faster return to spontaneous erections after surgery. But, follow-up periods used may have been short. In the available trials, follow-up was never longer than 12 months; although neuronal recovery after nsRP has been shown to take up to as long as 4 years (46, 47). Therefore, hard conclusion about the true effects on daily PDE5i on return to spontaneous erections after nsRP cannot be made yet. Looking at the other outcome measures, daily use of avanafil and tadalafil did show to improve rates of successful intercourse attempts after RP (8, 15). Moreover, on-demand use of vardenafil and tadalafil has shown to be effective in raising IIEF-EF domain scores after surgery (2, 15).

There may be a role for intraurethral alprostadil and intracavernosal injection therapy after nsRP; the available data point to positive effects of penile rehabilitation if regularly used. Unfortunately, not enough evidence is available to make clear recommendations on this point either.

Penile vibratory stimulation might be effective for the preservation and restoration of EF after nsRP in one trial, but more evidence is needed (20). Immunophilin ligands, especially tacrolimus, may be neuroprotective however, RCT did not affirm this (21). There is a controversy about the efficacy of shockwave therapy for ED in the literature. Some studies revealed improved outcomes for ED after RP when liESWT is used together with tadalafil, but these results were not statistically significant (22). Hyperbaric oxygen therapy showed no significant improvement on EF when used after RP (23).

Increased aerobic training after RP was shown to improve peripheral artery flow and restore the endothelial function (24), but it did not lead to better erections after surgery. Similarly, pelvic floor therapy is known to enhance blood flow to the penis, which is strongly correlated with EF (10). However, no statistically sound RCT have been conducted to demonstrate its positive effects on EF after RP (25).

After RT, sildenafil was shown to be an effective option for penile rehabilitation in several trials (12, 37, 40, 43); some could not demonstrate this positive effect (38).

On demand as well as daily PDE5i use after EBRT did improve patients' satisfaction with sexual intercourse in the first 2 years after RT (42). No studies have been conducted to evaluate the efficacy of PDE5i after RT in the long term. As it is well-known that ED often occurs several years after RT, more research is needed to point out whether PDE5i usage has protective effects 2–10 years after RT. Apart from the widely used treatment modalities for ED after RT, yoga interventions were shown to have positive effects on restoring EF after RT (36).

This study is limited due to a shortage of literature available when specifically looking for RCTs: only 24 of the 229 records could be included. There is a big inhomogeneity among studies, which makes it even more difficult to formulate clear recommendations.

However, this review presents an extensive overview of the different option for a big group of patients and points to the omissions in current literature.

When considering all aspects of EF recovery after RP and RT, other variables should be considered such as orgasmic dysfunction, climacturia, urinary incontinence, and the other adjuvant therapies that will decrease sexual function. To this regard, we believe that when evaluating sexual function, it is mandatory to take each aspect of sexuality into account and not just EF in its single form.

## Conclusion

This systematic review points to the positive effects of several non-medicinal therapy modalities that may contribute to recovery of spontaneous erections after RT and RP. Clear guidelines for penile rehabilitation after treatment for localized PCa are not easily provided based on current RCT available in literature. However, the importance of expectation management and provision of correct information for patients and their partners in the trajectory of Pca treatment cannot be overestimated.

Thus, until better evidence is available, results point to the positive effects of regular or daily use of a PDE5i directly after nsRP, so this treatment should not be withheld. PDE5i should be offered after a nsRP to all motivated patients with good erections prior to therapy.

In order to maximize chances of return to spontaneous erections or maintenance of erections after nsRP as well as RT, a combination of pelvic floor physiotherapy, vacuum device therapy, PVS, regular exercise and/or yoga may be recommended to be used together with pro-erectile medication. An holistic and multimodal approach may be the key to recovery of sexual function following RP or RT in patients with localized PCa.

## Data Availability Statement

The raw data supporting the conclusions of this article will be made available by the authors, without undue reservation.

## Author Contributions

All authors listed have made a substantial, direct and intellectual contribution to the work, and approved it for publication.

## Conflict of Interest

The authors declare that the research was conducted in the absence of any commercial or financial relationships that could be construed as a potential conflict of interest.
